# Integration of transient receptor potential canonical channels with lipids

**DOI:** 10.1111/j.1748-1716.2011.02311.x

**Published:** 2012-02

**Authors:** D J Beech

**Affiliations:** Faculty of Biological Sciences, Institute of Membrane and Systems Biology, University of LeedsLeeds, UK

**Keywords:** cation channel, lipid signalling, transient receptor potential

## Abstract

Transient receptor potential canonical (TRPC) channels are the canonical (C) subset of the TRP proteins, which are widely expressed in mammalian cells. They are thought to be primarily involved in determining calcium and sodium entry and have wide-ranging functions that include regulation of cell proliferation, motility and contraction. The channels are modulated by a multiplicity of factors, putatively existing as integrators in the plasma membrane. This review considers the sensitivities of TRPC channels to lipids that include diacylglycerols, phosphatidylinositol bisphosphate, lysophospholipids, oxidized phospholipids, arachidonic acid and its metabolites, sphingosine-1-phosphate, cholesterol and some steroidal derivatives and other lipid factors such as gangliosides. Promiscuous and selective lipid sensing have been detected. There appear to be close working relationships with lipids of the phospholipase C and A_2_ enzyme systems, which may enable integration with receptor signalling and membrane stretch. There are differences in the properties of each TRPC channel that are further complicated by TRPC heteromultimerization. The lipids modulate activity of the channels or insertion in the plasma membrane. Lipid microenvironments and intermediate sensing proteins have been described that include caveolae, G protein signalling, SEC14-like and spectrin-type domains 1 (SESTD1) and podocin. The data suggest that lipid sensing is an important aspect of TRPC channel biology enabling integration with other signalling systems.

There are seven mammalian genes encoding transient receptor potential canonical (TRPC) proteins and all of them except TRPC2 are expressed in humans (Flockerzi 2007, Nilius 2007, Venkatachalam & Montell 2007, Yildirim & Birnbaumer 2007, Abramowitz & Birnbaumer 2008). Like voltage-gated K^+^ channels, they are thought to form channels by gathering as a group of four around a central ion pore either using the same type (homomultimeric channels) or a mixture of transient receptor potentials (TRPs) (heteromultimeric channels). Initial studies suggested that TRPC1/4/5 and TRPC3/6/7 multimerize as exclusive subgroups, consistent with the observation that these TRPCs cluster in amino acid sequence comparisons. However, subsequent studies have suggested more flexibility (Strubing *et al.* 2003) and that other subtypes of TRP (e.g. TRPP2, TRPV4) may be incorporated (Tsiokas *et al.* 1999, Bai *et al.* 2008, Ma *et al.* 2010). The exact compositions of native TRPC-containing channels remain important unsolved problems.

The TRPC channels are permeable to the cations Ca^2+^, Na^+^ and K^+^. They are not voltage gated (i.e. they do not require a change in membrane potential to open), but they are often voltage sensitive (i.e. their activity is modulated by voltage). They may display significant constitutive activity (Dietrich *et al.* 2003, Nichols *et al.* 2007, Xu *et al.* 2008), but are most often stimulated or inhibited by a range of chemical or protein factors (Abramowitz & Birnbaumer 2008). They are emerging as polymodal ion channels that are sensitive to a multiplicity of activators and inhibitors, suggesting that they may serve as integrative sensors of complex chemical signals (Zeng *et al.* 2004, Abramowitz & Birnbaumer 2008). Importantly, although different cells express different relative amounts of TRPC, it seems that all mammalian cell types express most (if not all) of the TRPCs, suggesting that TRPC channels serve generic cell functions. For example, several TRPCs have been linked positively or negatively to cell migration, including an interesting reciprocal relationship between TRPC5 and TRPC6 through Rac1 and RhoA proteins respectively (Greka *et al.* 2003, Xu *et al.* 2006, Chaudhuri *et al.* 2008, Fabian *et al.* 2008, Tian *et al.* 2010).

Numerous specific functions of TRPCs in physiology and disease are starting to emerge, with examples that include roles of: TRPC1 in neointimal hyperplasia, cardiac hypertrophy, angiogenesis and saliva secretion (Jho *et al.* 2005, Kumar *et al.* 2006, Liu *et al.* 2007b, Seth *et al.* 2009, Yu *et al.* 2010); TRPC2 in pheromone sensation (Yildirim & Birnbaumer 2007); TRPC3 in pancreatitis, heart failure and NFKB activation (Kim *et al.* 2009, Kiyonaka *et al.* 2009, Smedlund *et al.* 2010); TRPC4 in gastrointestinal motility and blood pressure regulation (Freichel *et al.* 2001, Tsvilovskyy *et al.* 2009); TRPC5 in fear responses, regulation of matrix metalloprotease secretion from fibroblast-like synoviocytes and degranulation of mast cells (Ma *et al.* 2008, Xu *et al.* 2008, Riccio *et al.* 2009); and TRPC6 in familial focal segmental glomerulosclerosis, hypoxic pulmonary vasoconstriction, pulmonary hypertension, oesophageal cancer and angiogenesis (Winn *et al.* 2005, Weissmann *et al.* 2006, Hamdollah Zadeh *et al.* 2008, Shi *et al.* 2009).

This review addresses the topic of TRPC channel sensitivities to lipids as components of specific membrane environments or as active intracellular or intercellular signalling molecules. It explores the hypothesis that a function of TRPCs is to serve as integrators of lipid environments and signalling. An abridged summary is provided (Fig. 1), but it is a simplification of the available information and hence should be considered alongside the text below. The reader is referred to published review articles for general background on lipid structures, classification and signalling pathways, bilayer structures and lipid relevance to disease (Fahy *et al.* 2005, Maxfield & Tabas 2005, Wymann & Schneiter 2008, Marrink *et al.* 2009, Fukami *et al.* 2010, Sanchez-Mejia & Mucke 2010).

**Figure 1:**
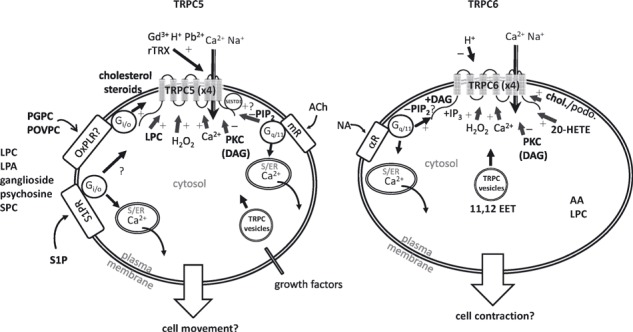
Abridged schematics for regulation of transient receptor potential canonical 5 (TRPC5) (left) and TRPC6 (right) channels by lipids in simplified mammalian cells. See the main text for details. Examples of regulation by other factors are included, but are not exhaustive. Abbreviations not provided in the main text are: Gd^3+^, gadolinium; Pb^2+^, lead; rTRX, reduced thioredoxin; OxPLR, putative oxidized phospholipid receptor (identity unknown); H_2_O_2_, hydrogen peroxide; chol., cholesterol; podo., podocin; AA, arachidonic acid. G_i/o_ and G_q/11_ are different types of GTP-binding proteins. The integral phospholipase C (PLC) and phospholipase A_2_ (PLA_2_) enzymes are not shown. TRPC6 may also be stimulated through receptor tyrosine kinase (Hamdollah Zadeh *et al.* 2008, Ge *et al.* 2009). Tian *et al.* (2010) should be consulted for the proposed distinction of TRPC5 coupling to cell movement and TRPC6 to contraction. Vesicular trafficking of TRPC5 has been described in response to growth factors (Bezzerides *et al.* 2004).

## Phosphatidylinositol (PI) phosphates

Phospholipase C (PLC) enzymes are stimulated by G proteins and receptor tyrosine kinases to generate key messengers such as inositol 1,4,5-trisphosphate (IP_3_) and diacyglycerols (DAGs) from phosphatidylinositol 4,5-bisphosphate (PIP_2_). PIP_2_ is not only the source of IP_3_ and DAGs, but also a key regulator of protein activity in its own right, as its local concentration depletes in response to receptor stimulation and target proteins contain PIP_2_-responsive elements. Phosphatidylinositol-3,4,5-trisphosphate (PIP_3_) is generated by the action of PI 3-kinase on PIP_2_, feeding into the Akt signalling pathway.

In heterologous expression studies, TRPC6 was found to be stimulated by PIP_2_ or PIP_3_, with PIP_3_ showing the highest affinity (Tseng *et al.* 2004, Kwon *et al.* 2007). It bound phosphoinositides directly at a site in the C terminus, competing with calmodulin (Kwon *et al.* 2007). Mutations in TRPC6 that decreased PIP_3_ binding suppressed channel activity, as did an Akt-PH domain that acted as a PIP_3_ sponge (Kwon *et al.* 2007). Other TRPC proteins were also found to bind phosphoinositides, especially TRPC1 (Kwon *et al.* 2007). PIP_2_ or PIP_3_ were subsequently found to stimulate endogenous channels containing TRPC1 in vascular smooth muscle cells (Saleh *et al.* 2009), whereas PIP_2_ inhibited endogenous TRPC6 or TRPC6/7 channels (Ju *et al.* 2010). Optimal receptor activation of TRPC6 was suggested to occur after depletion of PIP_2_ and generation of DAG (LarGe *et al.* 2009). The phosphatase and tensin homologue deleted on chromosome 10 (PTEN) phosphatase, which generates PIP_2_ from PIP_3_, was found to regulate TRPC6 surface expression independent of its phosphatase activity (Kini *et al.* 2010).

Phosphatidylinositol 4,5-bisphosphate inhibited TRPC4α but had no effect on TRPC4β channel (Otsuguro *et al.* 2008). Several other phosphoinositides had no effect, or stimulated TRPC4α. Evidence was provided for PIP_2_ interacting with the C terminus of TRPC4α and effects depended on the actin cytoskeleton and the postsynaptic density protein Drosophila disc large tumour suppressor and zona occludens-1 (PDZ)-binding motif of TRPC4 (Otsuguro *et al.* 2008). In one study, it was observed that PIP_2_ stimulated TRPC5 channel activity in excised inside-out patches, yet agents that caused PIP_2_ depletion had stimulatory effects on whole TRPC5 channel currents, as if PIP_2_ normally inhibited TRPC5 (Trebak *et al.* 2009). In another study, it was observed that PIP_2_ had no effect other than to slow the rate of TRPC5 channel desensitization following receptor activation (Kim *et al.* 2008).

A screen of a human aorta cDNA library revealed SESTD1 as a binding partner of TRPC4 and TRPC5 (Miehe *et al.* 2010). SESTD1 is a previously unrecognized protein that binds PI mono- and di-phosphates and phosphatidic acid, but not phosphatidylcholine, phosphatidylserine or PI (Miehe *et al.* 2010). Binding of PIP_2_ was shown to be Ca^2+^ dependent. SESTD1 was associated with the channels at the calmodulin/IP_3_ binding domain and suggested to be required for efficient receptor activation of the channels.

The data suggest that there are divergent and complex effects of PIP_2_ on TRPC channels. In several cases, the functional consequences require clarification and this situation is made more difficult by a evidence that TRPC heteromultimerization in native cells complicates the net effect of PIP_2_. Effects may occur through direct binding or intermediate proteins such as SESTD1.

## Diacyglycerols

Diacyglycerols are composed of two covalently linked fatty acids and may be formed from various sources, one of which is PIP_2_. Early studies searching for activators of TRPC channels identified DAGs as activators of the TRPC3/6/7 subgroup of TRPCs (Hofmann *et al.* 1999). TRPC2 was also activated by DAGs (Lucas *et al.* 2003). DAGs are now being used routinely as activators of these subclasses of TRPC channels. Various DAGs have been found to be effective, including 1-stearoyl-2-arachidonyl-sn-glycerol and the related 2,4-diacylphloroglucinols (Hofmann *et al.* 1999, Aires *et al.* 2007, Leuner *et al.* 2010). DAG activation of TRPC6 was not prevented by protein kinase C inhibitors, suggesting that it was independent of protein kinase C and had a relatively direct effect on the channel (Hofmann *et al.* 1999). On the basis of the computational analysis of amino acid sequences and mutagenesis studies, an N-terminal section of TRPC3/6/7 has been proposed as a DAG-sensing domain, although as a regulator vesicle fusion (van Rossum *et al.* 2008).

The concentrations of exogenous DAGs required to stimulate the channels are relatively high, but the effects are suggested to be relevant to endogenous DAGs because there is also activation by DAG lipase inhibitors (Hofmann *et al.* 1999). There is, nevertheless, evidence of synergism with IP_3_, potentially conferring greater sensitivity to DAG (Albert & Large 2003). Intriguingly, the effect of PIP_2_ on TRPC6/7, but not on TRPC6 channels was overcome by IP_3_ (Ju *et al.* 2010). The receptor activation of TRPC3/6/7 channels by agonists at G protein-coupled receptors is an effect that therefore arises, at least in part, because of G protein or receptor tyrosine kinase stimulation of PLCβ/γ leading to degradation and thus depletion of PIP_2_ and generation of DAGs and IP_3_, all of which impinge on the channels to varying degrees.

TRPC1 is not thought to be directly activated by DAGs, although TRPC is difficult to study on its own because trafficking to the plasma membrane is poor in the absence of other co-expressed factors (e.g. other TRP proteins). It has been activated by DAGs when co-expressed with TRPC3 (Lintschinger *et al.* 2000). It is, however, also described that TRPC1 was phosphorylated via protein kinase C (which is activated by DAGs) and that endogenous TRPC1-containing channels were stimulated, as a consequence (Ahmmed *et al.* 2004, Saleh *et al.* 2008). TRPC4 and 5 readily traffic to the plasma membrane but, in contrast to TRPC3/6/7, are not activated by DAG (Hofmann *et al.* 1999, Venkatachalam *et al.* 2003). There is, nevertheless, a suggestion that TRPC5 forms part of a DAG-activated channel with TRPC3 (Liu *et al.* 2007a). Furthermore, desensitization following receptor activation of TRPC4/5 occurred via protein kinase C-dependent phosphorylation (Venkatachalam *et al.* 2003, Zhu *et al.* 2005). Similarly, protein kinase C-inhibited TRPC3 (Trebak *et al.* 2005) and TRPC6 (Bousquet *et al.* 2010) suggest that DAGs have stimulatory (direct) and inhibitory (indirect via protein kinase C) effects on these channels.

Therefore, DAGs acutely and directly stimulate some of the TRPCs, but they also activate or inhibit TRPCs by triggering protein kinase C-dependent phosphorylation.

## Lysophospholipids

Lysophospholipids such as lysophosphatidylcholine (LPC) are generated by the enzymatic action of phospholipase A_2_ (PLA_2_) enzymes on phosphatidylcholine and other related substrates. LPC was first shown to be a stimulator of TRPC5 (Flemming *et al.* 2006). There is a partial insight into the mechanism of this effect. Chemically, the effect lacked head-group specificity because replacement of choline with inositol (to generate LPI) did not affect activity (Flemming *et al.* 2006). In contrast, the length of the carbon side chain was important, suggesting necessity of solubilization of the lysophospholipid in the lipid bilayer. As a result of this solubilization property, exogenous LPC has detergent effects on the lipid bilayers (hence ‘lyso’ indicating cell lysis). However, activation of TRPC5 occurred at low (subdetergent) concentrations of LPC and it was characterized by the distinctive current–voltage relationship (I–V) of TRPC5, showing that the effect reflected TRPC5 channel activity rather than non-specific bilayer disturbance.

Lysophosphatidylcholine is a ligand at certain G protein-coupled receptors (Ishii *et al.* 2004), but stimulation of TRPC5 by LPC did not require G protein signalling (Flemming *et al.* 2006). Furthermore, LPC activated TRPC5 in excised outside-out membrane patches in the absence of guanosine triphosphate (GTP), suggesting that it acted relatively directly at the channel. Consistent with negative data from convex membrane curvature experiments (Beech *et al.* 2009), LPC applied to the inner face of the lipid bilayer also activated TRPC5 (Flemming *et al.* 2006); that is, the effect of LPC on TRPC5 lacked polarity – acting similarly whether applied to the outside or inside of the membrane. This result is consistent with a model where membrane-spanning elements of TRPC5 contain a lipid interaction site that is accessible from either side of the membrane, conferring on the channel sensitivity to changes in lipid composition of the bilayer. Alternatively, TRPC5 activity may be influenced by specific membrane fluidity changes that occur with the introduction of LPC.

TRPC6-containing channels were found to be stimulated by LPC in endothelial cells (Chaudhuri *et al.* 2008). The I–V of the stimulated current lacked the distinctive rectification of TRPC channels, but responses were reduced when TRPC6 expression was suppressed or prevented, suggesting TRPC6 was involved but not alone. Compelling biochemical evidence was presented for forward trafficking of TRPC5 in response to LPC-evoked TRPC6-dependent Ca^2+^ entry. In these cells, TRPC5 expression at the plasma membrane was initially low, which might explain why there was no obvious stimulation of TRPC5 in the absence of TRPC6.

Stimulation of TRPC channels by LPC has biological importance in endothelial cell migration (Chaudhuri *et al.* 2008). It may also have wider importance. Human monocytes, for example, showed Ca^2+^ entry in response to LPC that was independent of G protein and PLC signalling and dependent on LPC carbon chain length (Yun *et al.* 2004). Human monocytes express TRPC5 and other TRPCs (Liu *et al.* 2007a). The pharmacological profile of the LPC-activated current in monocytes was similar to that of TRPC6 (Schilling & Eder 2009). A role of TRPC activation by LPC has been suggested in erectile dysfunction (So *et al.* 2005). Moreover, LPC is a major component of oxidized low-density lipoprotein (oxLDL), which may explain the Ca^2+^ influx and apoptosis induced by oxLDL in vascular smooth muscle cells (Ingueneau *et al.* 2008). Various endogenous non-selective cationic channels have been found to be stimulated by LPC, but it is not clear whether they are explained by TRPC channels (Smani *et al.* 2004, Schilling & Eder 2009).

Therefore, lysophospholipids stimulate TRPC channels, apparently relatively directly. The effects are relevant to endogenous concentrations of lysophospholipids and may be important in wide-ranging biological phenomena, both in terms of intracellular and extracellular signalling.

## Oxidized phospholipids

1-Palmitoyl-2-arachidonoyl-sn-glycero-3-phosphorylcholine is a common component of cell membranes. Its susceptibility to oxidation leads to bioactive oxidation products called oxidized phospholipids, which include 1-palmitoyl-2-oxovaleroyl-phosphatidylcholine (POVPC) and 1-palmitoyl-2-glutaroyl-phosphatidylcholine (PGPC). These lipids constitute a diverse family of signalling lipids that accumulate during oxidative stress, apoptosis and necrosis, and are often associated with inflammatory conditions such as rheumatoid arthritis and atherosclerosis. There are also suggestions of physiological roles for oxidized phospholipids that include pattern recognition in innate immunity. Although the importance of oxidized phospholipids is increasingly established, the initial reception and signalling mechanisms have been poorly understood. A recent study revealed that these lipid factors are stimulators of TRPC5 or TRPC5-containing channels (Al-Shawaf *et al.* 2010).

Low micromolar concentrations of PGPC and POVPC stimulated TRPC5 exogenously expressed in human embryonic kidney (HEK) 293 cells (Al-Shawaf *et al.* 2010). Relevance to endogenous TRPC5-containing channels was found in vascular smooth muscle cells where the oxidized phospholipids evoked TRPC1/5 channel activity without causing Ca^2+^ release. The effect was functionally relevant to cell migration. Surprisingly, given the chemical similarity to LPC, the actions of PGPC and POVPC depended almost completely on G-protein (G_i/o_) signalling (Al-Shawaf *et al.* 2010). Previously identified G protein-coupled receptors for oxidized phospholipids were not involved, suggesting that the effects occurred via a previously unrecognized receptor or independently of receptors, but nevertheless requiring G protein function. In our experience, these oxidized phospholipids are amongst the best TRPC5 (or TRPC1/5) activators, having an advantage that the activation occurs without complications from Ca^2+^ release (Al-Shawaf *et al.* 2010).

## Arachidonic acid and its metabolites

Arachidonic acid is a polyunsaturated fatty acid of lipid bilayers that is generated by phospholipase enzymes and is the precursor for many active metabolites. There are reports that TRPC channels are modulated by arachidonic acid and some of its metabolites. Basora *et al.* (2003) reported direct activation of TRPC6 by arachidonic acid and its metabolite 20-hydroxyeicosatetraenoic acid (20-HETE). The I–V of the stimulated current resembled that of TRPC6 only at high 20-HETE concentrations and, surprisingly, no Ca^2+^ entry was evoked by 20-HETE despite the fact that current was observed (Basora *et al.* 2003). Inoue *et al.* (2009) also reported TRPC6 stimulation by 20-HETE, in this case with a distinct TRPC6 I–V. Furthermore, dependence of hypotonic- or 2,4,6-trinitrophenol-stimulated TRPC6 on cytosolic PLA_2_ activity was identified (Inoue *et al.* 2009). Relationships of TRPC channels with arachidonic acid metabolites have also been suggested by other studies. Ben-Amor *et al.* (2006) reported block by anti-TRPC1 antibody of Ca^2+^ entry evoked by 5,6-epoxyeicosatrienoic acid (5,6-EET) in human platelets and Fleming *et al.* (2007) reported surface trafficking of TRPC6 in response to 11,12-EET and pulmonary vasoconstriction evoked by 11,12-EET was less in lungs from TRPC6 gene-disrupted mice. A stable urea EET analogue has been suggested to act through TRPC channel modulation (Liu *et al.* 2011) and 15-HETE has been observed to stimulate TRPC1 expression (Li *et al.* 2010).

Wu *et al.* (2002) suggested contribution of endogenous TRPC4 to arachidonic acid-evoked Ca^2+^ entry in HEK 293 cells. However, we have found no activation by arachidonic acid of the related TRPC5 channel overexpressed in HEK 293 cells (Flemming *et al.* 2006, Beech *et al.* 2009). TRPC5 is stimulated by the arachidonic acid metabolite prostaglandin E2, but acts through the E-type prostaglandin-1 (EP1) G protein-coupled receptor (Tabata *et al.* 2002). TRPC7 has been suggested to be required for induction of apoptosis by prostaglandin E2 in leukaemia cells (Foller *et al.* 2006).

In summary, arachidonic acid metabolites have importance as stimulators of TRPC channels, most notably of TRPC6 channels.

## Sphingosine-1-phosphate

Sphingosine-1-phosphate (S1P) is generated from sphingosine, which derives from sphingomyelin, a constituent lipid of microdomains in the plasma membrane. TRPC5 was stimulated by S1P (Xu *et al.* 2006). S1P applied to the intracellular surface stimulated TRPC5 in inside-out membrane patches. TRPC5 was, therefore, suggested to be an intracellular target for S1P but without known physiological importance. Potentially related is the observation that S1P bound to a putative TRPC3-PLCγ1 intermolecular domain that also interacted with PI phosphates, although the functional relevance of this binding was not determined (van Rossum *et al.* 2005). Unlike for LPC, the extracellular effect of S1P on TRPC5 occurred via a G-protein (G_i/o_) signalling pathway (Xu *et al.* 2006), further illustrating the significance of TRPC activation via receptors that have lipids as their ligands. S1P receptors are widely expressed, including HEK 293 cells often used for TRPC5 overexpression. S1P had no effect on TRPC5 studied in excised outside-out patches without GTP in the pipette, showing that S1P (unlike LPC) had no direct extracellular effect on TRPC5. The extracellular S1P effect on TRPC5 was found to be functionally important in cell motility (Xu *et al.* 2006). Therefore, S1P is an example of a lipid factor that activates TRPC channels via a G-protein signalling pathway (Xu *et al.* 2006), in some ways similar to the action of oxidized phospholipids (Al-Shawaf *et al.* 2010), but involving Ca^2+^ release also. Suggested intracellular actions of S1P could be biologically important, but remain relatively little explored.

## Cholesterol and derivatives (steroids)

Cholesterol is a constituent sterol lipid of the plasma membrane. Its depletion with methyl-β-cyclodextrin has been shown to suppress store-operated Ca^2+^ signals and ionic current linked to TRPC1 (Bergdahl *et al.* 2003, Kannan *et al.* 2007, Alicia *et al.* 2008). Similarly, cholesterol loading of cells was found to have a positive effect on signals relating to TRPC3 (Graziani *et al.* 2006). TRPC1 has been associated with cholesterol-containing caveolae and other lipid rafts (Lockwich *et al.* 2000) and suggested to function as a component of store-operated channels only when linked to stromal interaction molecule-1 (STIM1) in lipid rafts (Alicia *et al.* 2008). Several studies have linked TRPC1 with caveolins (Lockwich *et al.* 2000, Bergdahl *et al.* 2003, Remillard & Yuan 2006, Ingueneau *et al.* 2008). An elegant study (Huber *et al.* 2006) showed enhancement of TRPC6 by the cholesterol-binding protein podocin, dependent on cholesterol binding by podocin which localizes specifically to the slit diaphragm of the kidney and is present at the inner leaflet of the bilayer. Cholesterol depletion with methyl-β-cyclodextrin inhibited the effect of podocin on TRPC6.

Cholesterol is the precursor for steroid hormones such as the neuroactive steroids, which are synthesized in the brain, adrenal glands and gonads (Compagnone & Mellon 2000). Examples of neuroactive steroids are pregnenolone sulphate and allopregnanolone. Specific types of neuroactive steroid have inhibitory actions in TRPC5, strengthening the emerging idea that TRP channels have unique steroid-sensing capabilities (Wagner *et al.* 2008, Majeed *et al.* 2010). TRPC5 was found to be negatively modulated via a rapid non-genomic mechanism (Majeed *et al.* 2011). The channels were inhibited by pregnenolone sulphate, pregnanolone (or its β-sulphated form), progesterone or dihydrotestosterone. There was a small effect of 17β-oestradiol, but no effect of pregnenolone, allopregnanolone or cortisol. Rapid and reversible effects of progesterone were shown in excised membrane patches. Sensitivity to pregnanolone, but not its stereo-isomer allopregnanolone, suggested the existence of a specific binding site. Endogenous TRPC1/5 channels were also inhibited by progesterone, albeit at a relatively high concentration. A prior study suggested that TRPC2, which is not expressed in humans, is activated by sulphated steroids from the urine, with importance for odour sensation of rodents (Nodari *et al.* 2008).

The data suggest dependence of TRPC channels on cholesterol and modulation of TRPC function by localization to lipid rafts. Furthermore, it is emerging that TRPC channels show highly specific and potentially unique steroid-sensing capability leading to inhibition of channel function.

## Gangliosides and other lipid factors

Using an intracellular Ca^2+^ assay for HEK 293 cells conditionally overexpressing TRPC5, we investigated additional lipid factors as potential acute activators (Beech *et al.* 2009). Several lysophospholipids were activators, including the important signalling lipid lysophosphatidic acid, but not lysophosphatidylethanolamine or phosphatidylcholine. Platelet-activating factor (PAF) and lyso-PAF (which is inactive at PAF receptors) were activators at 3 *μ*m concentration; both are chemically similar to LPC. Sphingosine, sphingomyelin, ceramide and ceramide-1-phosphate (C1P) were not stimulators of the channels but sphingosylphosphorylcholine was, by contrast, a strong activator. Cerebrosides, sulphatides and anandamide (an arachidonic acid metabolite) failed to activate but gangliosides and psychosine were modest activators. Gangliosides are glycosphingolipids containing sialic acid. It was found that crosslinking of the monosialosyl of gangliotetraose (GM1) ganglioside with multivalent ligands stimulated endogenous TRPC5-containing channels via α5β1 integrin (Wu *et al.* 2007); the effect was important in neuronal growth cone formation. We did not find a stimulatory effect of C1P on TRPC5, but it was recently reported that ceramide kinase and TRPC1 colocalize in cavealae (Hinkovska-Galcheva *et al.* 2008), raising the possibility that endogenous TRPC complexes are sensitive to C1P. Furthermore, in a human leukaemia T-cell line, Ca^2+^ entry evoked by Δ^9^-tetrahydrocannabinoid (a lipid-soluble plant-derived cannabinoid) was suppressed when TRPC1 was downregulated by RNA interference (Rao & Kaminski 2006); the effect occurred through cannabinoid G protein-coupled receptors. Therefore, there is an emerging breadth to the spectrum of lipids that modulate TRPC channels but also evidence of specificity.

## Relationships to receptor agonists, membrane stretch and anaesthetics

As indicated above, common downstream effects of agonist binding to receptors are activation of PLC and PLA_2_ enzymes, which affect local concentrations of PIP_2_, DAGs, arachidonic acid metabolites, etc. Therefore, a consequence of lipid sensitivity of TRPC channels is that they are modulated, often positively, by a plethora of G protein-coupled or tyrosine kinase receptor agonists. Related to such effects may be the reported stimulatory effects of membrane deformation or stretch on the TRPC channels and the suggested relevance to myogenic tone in arteries (Welsh *et al.* 2002, Maroto *et al.* 2005, Gomis *et al.* 2008). Myogenic tone, for example, is associated with elevated levels of DAG, arachidonic acid metabolites and oxidative stress factors such as hydrogen peroxide (Hill *et al.* 2009). Stretch activation of TRPC6 has been suggested to arise because of sensitivity of G protein-coupled receptors and associated signalling pathways to membrane deformation, leading to downstream effects on TRPC6 activity (Mederos y Schnitzler *et al.* 2008, Inoue *et al.* 2009).

In part, anaesthetics modulate ion channel function by disturbing the lipid bilayer. Therefore, lipid sensitivity of TRPC channels may confer sensitivity to anaesthetics, as occurs with other ion channels. TRPC5 was found to be sensitive to general anaesthetics with the dominant net effect being inhibition of channel function (Bahnasi *et al.* 2008). The study included the surprising finding that TRPC5 stimulated by LPC was resistant to the intravenous anaesthetic propofol, whereas TRPC5 stimulated by gadolinium was strongly inhibited. It was suggested that propofol may not directly inhibit TRPC5, but instead compromised a signalling pathway that was necessary for TRPC5 activation by the lanthanide (Bahnasi *et al.* 2008). The data suggest a complex relationship between anaesthetics and TRPC5. It is not known if other TRPC channels are sensitive to anaesthetics.

## Summary and conclusions

Transient receptor potential canonicals (TRPCs) have emerged as a class of proteins that form lipid-sensing cationic channels. Understanding remains elementary but, in some instances, we may start to consider them as lipid ionotropic receptors or lipid sensors through intermediate proteins. It should be recognized, nevertheless, that TRPC channels also exhibit constitutive activity (Dietrich *et al.* 2003, Nichols *et al.* 2007, Xu *et al.* 2008) and can be modulated by non-lipid factors that include extracellular acid, toxic metal ions, intracellular Ca^2+^, hydrogen peroxide and redox proteins (Schaefer *et al.* 2000, Strubing *et al.* 2001, Shi *et al.* 2004, Zeng *et al.* 2004, Hui *et al.* 2006, Semtner *et al.* 2007, Xu *et al.* 2008, Graham *et al.* 2010, Naylor *et al.* 2011). Although TRPC channels are responsive to lipid factors, they may not depend on them.

Figure 1 gives an abridged summary of knowledge of lipids and additional factors that modulate TRPC5 or TRPC6, providing comparisons for these two examples of TRPCs that have been studied relatively intensely. However, it should be recognized that the diagrams overly simplify the situation and hence they should be studied alongside the main text of this article and the original research publications. Although other TRPCs may show similar characteristics (e.g. TRPC4 like TRPC5, TRPC3 like TRPC6), there are also important differences, and incorporation of TRPC1 and other heteromultimeric assemblies may have a significant impact. Nevertheless, some impressions can be gained from the diagrams: the clearest distinctions between the channels are the activation of TRPC6 but not TRPC5 by DAG and arachidonic acid metabolites; in both cases there is evidence of promiscuity but also selectivity; the lipid profiles are consistent with intricate relationships of TRPCs with PLC and PLA_2_ enzymes; and, although not absolute, it is emerging that TRPC5 may be more associated with cell migration and proliferation, whereas TRPC6 is more associated with cell contraction and stability.

It seems clear that TRPC channels are capable of sensing various important lipids, enabling them to respond to these lipids as part of signalling events or to integrate with dynamic lipid environments of physiological or pathological contexts. Despite the technical difficulties of such studies, further investigation of TRPC modulation by lipids will be important. In many cases, knowledge of the lipid-sensing profile of a TRPC channel is limited or there is information only about a TRPC overexpressed in a cell line rather than endogenously as a heteromultimeric complex. In most cases, the mechanism of action of the lipid is unknown or superficially understood. The potential for synergy between actions of lipids and other factors has been underexplored.
